# The genome sequence of *Inga laurina* (Sw.) Willd.

**DOI:** 10.12688/wellcomeopenres.23057.1

**Published:** 2024-10-07

**Authors:** Rowan J. Schley, R. Toby Pennington, Alex D. Twyford, Kyle G. Dexter, Catherine Kidner, Todd P. Michael, Wellcome Sanger Institute Tree of Life Management, Samples and Laboratory team, Wellcome Sanger Institute Scientific Operations: Sequencing Operations, Wellcome Sanger Institute Tree of Life Core Informatics team, Tree of Life Core Informatics collective

**Affiliations:** 1 University of Exeter, Exeter, England, UK; 2 Royal Botanic Garden Edinburgh, Edinburgh, Scotland, UK; 3 The University of Edinburgh, Edinburgh, Scotland, UK; 4 University of Turin, Turin, Italy; 5 Salk Institute for Biological Studies, La Jolla, California, USA; 6 University of California San Diego, San Diego, California, USA; 7 San Diego Botanical Garden, San Diego, California, USA

**Keywords:** Inga laurina, genome sequence, chromosomal, Fabales

## Abstract

We present a genome assembly from an individual of *Inga laurina* (Streptophyta; Magnoliopsida; Fabales; Fabaceae). The genome sequence has a total length of 899.60 megabases. Most of the assembly is scaffolded into 13 chromosomal pseudomolecules, supporting the individual being an autotetraploid with 2 *n*=4 *x*=52. The mitochondrial and plastid genome assemblies have lengths of 1,261.88 kilobases and 176.27 kilobases, respectively. Gene annotation of this assembly on Ensembl identified 33,101 protein-coding genes.

## Species taxonomy

Eukaryota; Viridiplantae; Streptophyta; Streptophytina; Embryophyta; Tracheophyta; Euphyllophyta; Spermatophyta; Magnoliopsida; Mesangiospermae; eudicotyledons; Gunneridae; Pentapetalae; rosids; fabids; Fabales; Fabaceae; Caesalpinioideae; mimosoid clade; Ingeae; *Inga*; *Inga laurina* (Sw.) Willd. (NCBI:txid487684).

## Background

The neotropical rainforest tree genus *Inga* Mill. (Fabaceae) is a ubiquitous and characteristic component of the species-rich flora of the tropical Americas. *Inga* typifies the rapid evolutionary radiations that generated most neotropical tree diversity, exhibiting the highest diversification rate of any tree genus in the Amazon ( Baker *et al.*, 2014; Richardson *et al.*, 2001). *Inga laurina* (Sw.) Willd. is the most broadly-distributed *Inga* species, ranging from north-eastern Mexico through Central and South America to Paraguay and Argentina, also occurring in both the Greater and Lesser Antilles ( Pennington, 1997). It is found at elevations of 0–1500 m, mostly in rainforest, but is particularly adaptable in terms of rainfall, and so is occasionally found in drier habitats such as the Cerrado savanna of Brazil or the dry scrubland of the Antilles.

*Inga laurina*, like all tropical rainforest tree species, is subject to high levels of insect herbivory and therefore has evolved several defensive strategies to deter herbivores. Specifically, *I. laurina* possesses extra-floral nectaries on its leaf midribs for attracting ants that defend the plant against herbivores, as well as defending itself chemically by over-expressing the amino acid tyrosine in its young leaves ( Kahl *et al.*, 2019). *Inga* species such as *I. laurina* are nitrogen fixers and also produce fruits with seeds covered by a sweet, white seed coat (sarcotesta) that is widely used as food and livestock fodder ( Pennington, 1997). Accordingly, *Inga laurina* is sometimes used in agroforestry settings for nitrogen fixation ( da Silva *et al.*, 2014) or horticulturally, and the fruits are occasionally gathered from the wild for use by local communities ( Lorenzi, 2002; Lorenzi *et al.*, 2006). However, it is not as widely used as other *Inga* species (e.g., *Inga edulis* or *I. macrophylla*).

*Inga laurina* displays broad morphological variation, and it has been suggested that it may comprise several undescribed cryptic species ( Dexter *et al.*, 2010). Further, the species can be readily confused with other relatively non-descript *Inga* species with two pairs of glabrous, elliptic leaflets. The sample sequenced here, originally collected in Brazil but grown at Royal Botanic Garden Edinburgh (RBGE), is a tetraploid (2 *n*=4 *x*=52) ( Hanson, 1995), which is most typical for the species, although diploid populations of *Inga laurina* are also known in other regions of Brazil (2 *n*=2 *x*=26) ( Figueiredo *et al.*, 2014).

Here we present one of three chromosomally complete, annotated genome sequences for *Inga*. These genomes are the first for the genus and we believe they will be a crucial resource for future work. This *Inga laurina* genome will be useful for a broad range of fields, given the agricultural utility of *Inga* and its prominent role as a model group for understanding the ecology and evolution of tropical rainforest floras. Future avenues of study may include genomic characterisation of enzymes underlying antifungal compounds produced by *I. laurina* seeds and bark ( de Moura Martins *et al.*, 2020; Macedo *et al.*, 2016), those underlying production of trypsin inhibitors and saponins ( Cruz *et al.*, 2016; de Moura Martins *et al.*, 2020), and those related to nutritional development ( Martins *et al.*, 2024). In addition, nitrogen-fixing activity and symbionts ( da Silva *et al.*, 2014) and exploration of polyploidisation history in tropical trees would be fruitful avenues of future research.

## Genome sequence report

**Figure 1.**
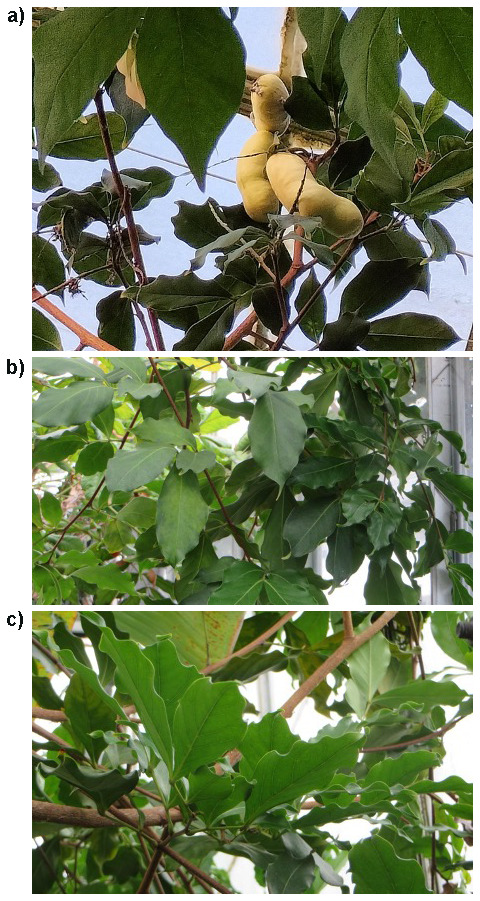
Photographs of the *Inga laurina* (drIngLaur1) specimen used for genome sequencing collected from the living collection at Royal Botanic Garden Edinburgh, detailing fruit ( **a**) and leaves ( **b** and **c**).

The genome of a specimen of *Inga laurina* ( Figure 1) was sequenced using Pacific Biosciences single-molecule HiFi long reads, generating a total of 30.32 Gb (gigabases) from 2.41 million reads, providing approximately 30-fold coverage. Primary assembly contigs were scaffolded with chromosome conformation Hi-C data, which produced 87.06 Gb from 576.53 million reads, yielding an approximate coverage of 97-fold. Specimen and sequencing information is summarised in Table 1.

**Table 1.** Specimen and sequencing data for *Inga laurina*.

**Project information**			
**Study title**	Inga laurina		
**Umbrella BioProject**	PRJEB65690		
**Species**	*Inga laurina*		
**BioSample**	SAMEA110107569		
**NCBI taxonomy ID**	487684		
Specimen information			
**Technology**	**ToLID**	**BioSample accession**	**Organism part**
**PacBio long read sequencing**	drIngLaur1	SAMEA110107570	Leaf
**Hi-C sequencing**	drIngLaur1	SAMEA110107576	Leaf
**RNA sequencing**	drIngLaur1	SAMEA110107577	Leaf
Sequencing information			
**Platform**	**Run accession**	**Read count**	**Base count (Gb)**
**Hi-C Illumina NovaSeq 6000**	ERR12035271	6.92e+08	104.45
**Hi-C Illumina NovaSeq 6000**	ERR12035272	5.77e+08	87.06
**PacBio Sequel IIe**	ERR12015746	2.38e+06	28.48
**PacBio Sequel IIe**	ERR12015747	2.41e+06	30.32
**RNA Illumina NovaSeq 6000**	ERR12245593	8.88e+07	13.4
**RNA Illumina NovaSeq 6000**	ERR12245594	9.60e+07	14.5
**RNA Illumina NovaSeq 6000**	ERR12245592	7.03e+07	10.61

**Figure 2.**
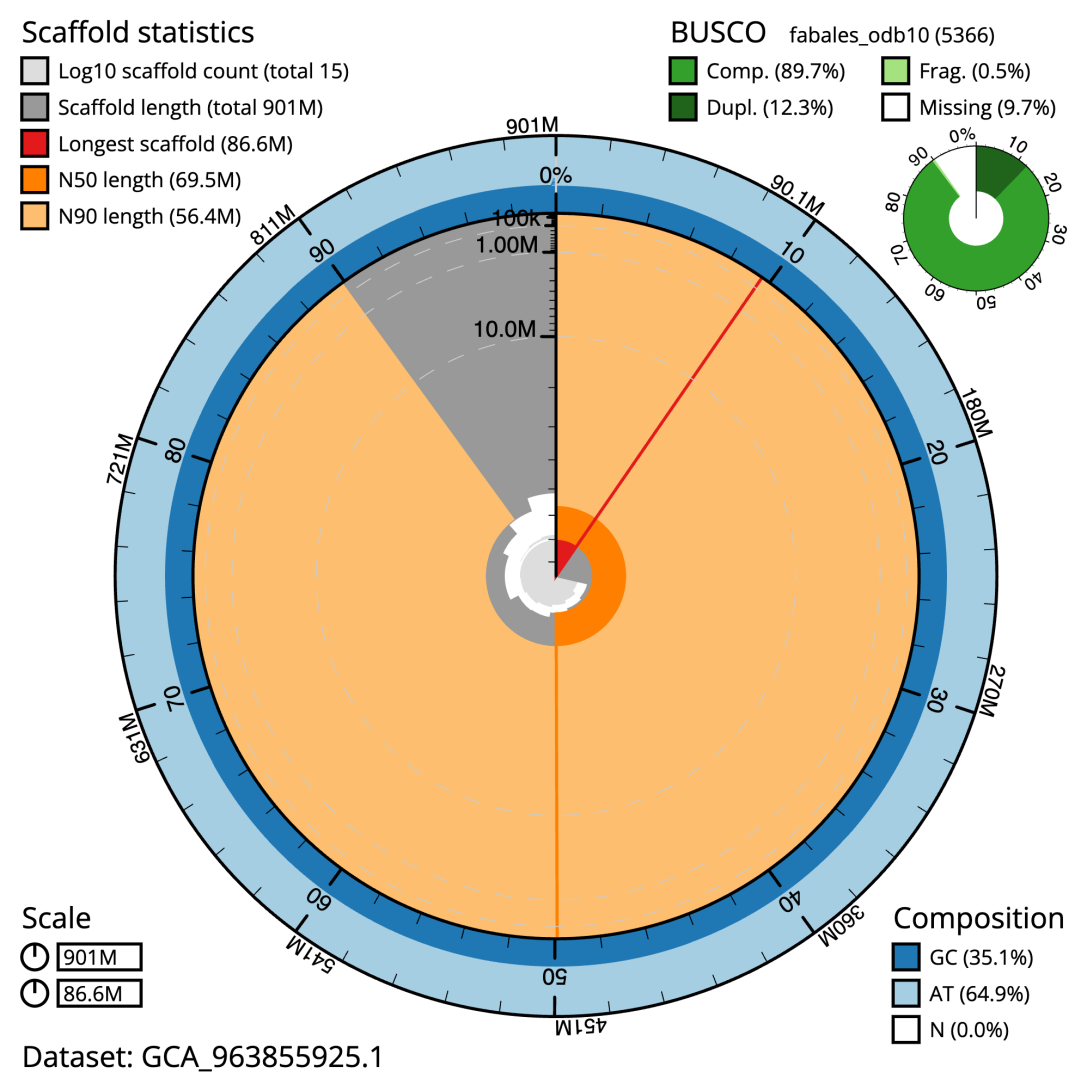
Genome assembly of *Inga laurina*, drIngLaur1.1: metrics. The BlobToolKit snail plot shows N50 metrics and BUSCO gene completeness. The main plot is divided into 1,000 size-ordered bins around the circumference with each bin representing 0.1% of the 901,002,922 bp assembly. The distribution of scaffold lengths is shown in dark grey with the plot radius scaled to the longest scaffold present in the assembly (86,578,616 bp, shown in red). Orange and pale-orange arcs show the N50 and N90 scaffold lengths (69,475,142 and 56,366,859 bp), respectively. The pale grey spiral shows the cumulative scaffold count on a log scale with white scale lines showing successive orders of magnitude. The blue and pale-blue area around the outside of the plot shows the distribution of GC, AT and N percentages in the same bins as the inner plot. A summary of complete, fragmented, duplicated and missing BUSCO genes in the fabales_odb10 set is shown in the top right. An interactive version of this figure is available at https://blobtoolkit.genomehubs.org/view/GCA_963855925.1/dataset/GCA_963855925.1/snail.

**Figure 3.**
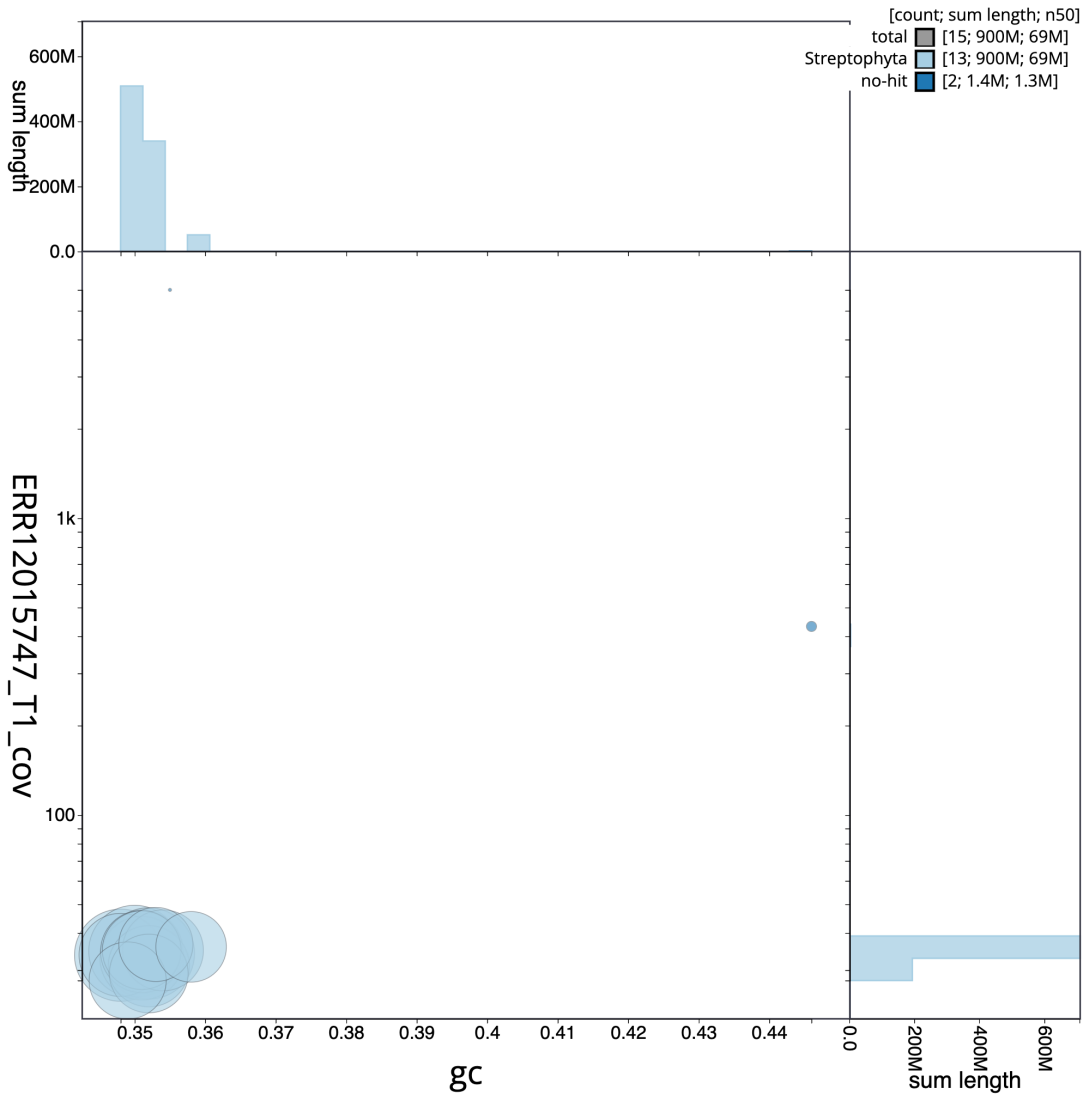
Genome assembly of *Inga laurina*, drIngLaur1.1: BlobToolKit GC-coverage plot. Scaffolds are coloured by phylum. Circles are sized in proportion to scaffold length. Histograms show the distribution of scaffold length sum along each axis. An interactive version of this figure is available at https://blobtoolkit.genomehubs.org/view/GCA_963855925.1/dataset/GCA_963855925.1/blob.

**Figure 4.**
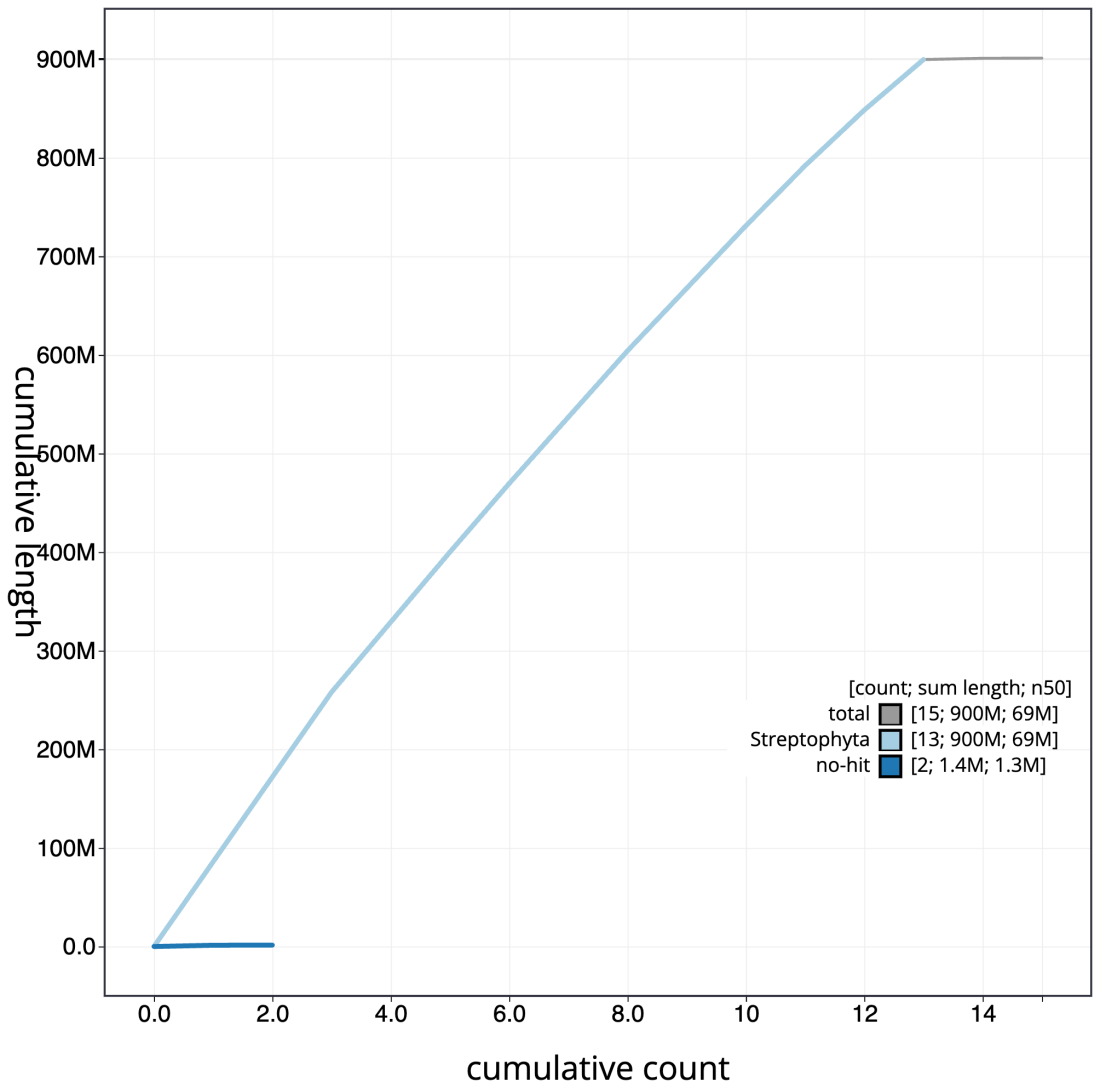
Genome assembly of *Inga laurina*, drIngLaur1.1: BlobToolKit cumulative sequence plot. The grey line shows cumulative length for all scaffolds. Coloured lines show cumulative lengths of scaffolds assigned to each phylum using the buscogenes taxrule. An interactive version of this figure is available at https://blobtoolkit.genomehubs.org/view/GCA_963855925.1/dataset/GCA_963855925.1/cumulative.

**Figure 5.**
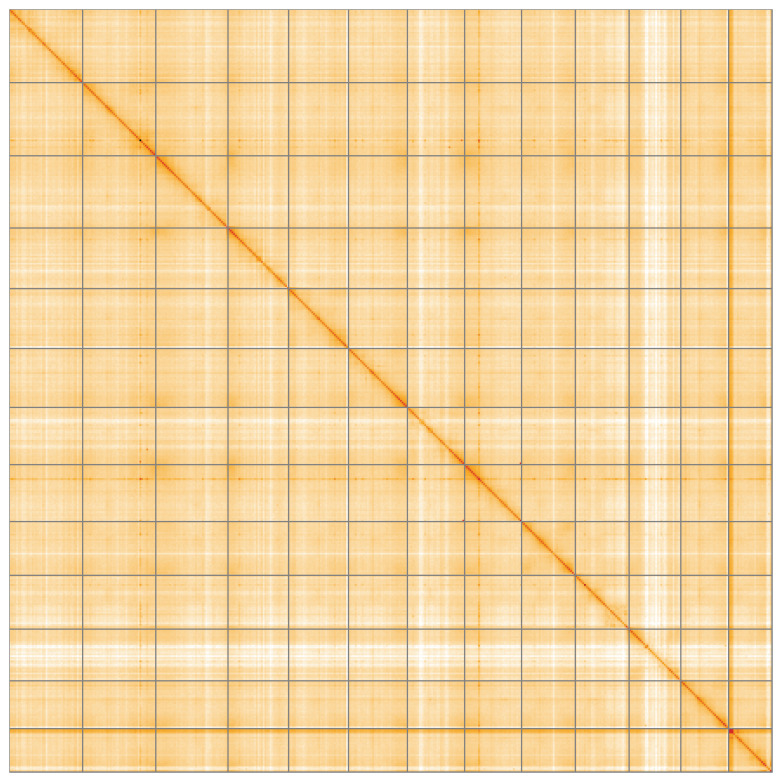
Genome assembly of *Inga laurina*, drIngLaur1.1: Hi-C contact map of the drIngLaur1.1 assembly, visualised using HiGlass. Chromosomes are shown in order of size from left to right and top to bottom. An interactive version of this figure may be viewed at https://genome-note-higlass.tol.sanger.ac.uk/l/?d=SQykJr0hQwSzxP3-QpQk9Q.

Manual assembly curation corrected 260 missing joins or mis-joins and 6348 haplotypic duplications, reducing the assembly length by 75.64%, and increasing the scaffold N50 by 16.62%. The final assembly has a total length of 899.60 Mb in 13 sequence scaffolds with a scaffold N50 of 69.5 Mb ( Table 2) with 651 gaps. The snail plot in Figure 2 provides a summary of the assembly statistics, while the distribution of assembly scaffolds on GC proportion and coverage is shown in Figure 3. The cumulative assembly plot in Figure 4 shows curves for subsets of scaffolds assigned to different phyla. Most (99.84%) of the assembly sequence was assigned to 13 chromosomal-level scaffolds. Chromosome-scale scaffolds confirmed by the Hi-C data are named in order of size ( Figure 5; Table 3). The primary assembly contains a manually phased (based on Hi-C signal) single haplotype version of the tetraploid *Inga laurina* genome. All unplaced content and the three alternative haplotypes are placed in the alternate assembly file. The Hi-C contact map for the single primary haplotype has some ambiguous regions due to structural variation between the haplotypes. The mitochondrial and plastid genomes were also assembled and can be found as contigs within the multifasta file of the genome submission.

**Table 2.** Genome assembly data for *Inga laurina*, drIngLaur1.1.

Genome assembly		
Assembly name	drIngLaur1.1	
Assembly accession	GCA_963855925.1	
*Accession of alternate haplotype*	*GCA_963855915.1*	
Span (Mb)	899.60	
Number of contigs	666	
Contig N50 length (Mb)	2.2	
Number of scaffolds	13	
Scaffold N50 length (Mb)	69.5	
Longest scaffold (Mb)	86.58	
Assembly metrics *		*Benchmark*
Consensus quality (QV)	66.3	*≥ 50*
*k*-mer completeness	100.0%	*≥ 95%*
BUSCO **	C:89.5%[S:77.3%,D:12.2%], F:0.7%,M:9.8%,n:5,366	*C ≥ 95%*
Percentage of assembly mapped to chromosomes	99.84%	*≥ 95%*
Organelles	Mitochondrial genome: 1261.88 kb Plastid genome: 176.27 kb	*complete single alleles*
Genome annotation at Ensembl		
Number of protein-coding genes	33,101	
Number of non-coding genes	12,113	
Number of gene transcripts	65,667	

* Assembly metric benchmarks are adapted from column VGP-2020 of “Table 1: Proposed standards and metrics for defining genome assembly quality” from Rhie *et al.* (2021). ** BUSCO scores based on the fabales_odb10 BUSCO set using version 5.4.3. C = complete [S = single copy, D = duplicated], F = fragmented, M = missing, n = number of orthologues in comparison. A full set of BUSCO scores is available at https://blobtoolkit.genomehubs.org/view/Inga_laurina/dataset/GCA_963855925.1/busco.

**Table 3.** Chromosomal pseudomolecules in the genome assembly of *Inga laurina*, drIngLaur1.

INSDC accession	Name	Length (Mb)	GC%
OY979701.1	1	86.58	35.0
OY979702.1	2	86.25	35.0
OY979703.1	3	85.18	35.0
OY979704.1	4	71.48	35.0
OY979705.1	5	70.62	35.0
OY979706.1	6	69.48	35.0
OY979707.1	7	67.47	35.0
OY979708.1	8	67.12	35.5
OY979709.1	9	63.44	35.0
OY979710.1	10	63.43	35.0
OY979711.1	11	60.94	35.0
OY979712.1	12	56.37	35.5
OY979713.1	13	51.21	36.0
OY979714.1	MT	1.26	44.5
OY979715.1	Pltd	0.18	35.5

The estimated Quality Value (QV) of the final assembly is 66.3 with *k*-mer completeness of 100.0%, and the assembly has a BUSCO v5.4.3 completeness of 89.5% (single = 77.3%, duplicated = 12.2%), using the fabales_odb10 reference set ( *n* = 5,366).

Metadata for specimens, BOLD barcode results, spectra estimates, sequencing runs, contaminants and pre-curation assembly statistics are given at https://links.tol.sanger.ac.uk/species/487684.

## Genome annotation report

The *Inga laurina* genome assembly (GCA_963855925.1) was annotated at the European Bioinformatics Institute (EBI) on Ensembl Rapid Release. The resulting annotation includes 65,667 transcribed mRNAs from 33,101 protein-coding and 12,113 non-coding genes ( Table 2; https://rapid.ensembl.org/Inga_laurina_GCA_963855925.1/Info/Index). The average transcript length is 3,508.99. There are 1.45 coding transcripts per gene and 4.91 exons per transcript.

## Methods

### Sample acquisition

A specimen of *Inga laurina* (specimen ID SAN2000549, ToLID drIngLaur1) was collected on 2021-09-09 from the wet tropics glasshouse at the Royal Botanic Garden Edinburgh, Scotland, UK. The specimen was collected by Rowan Schley (University of Exeter). The original individual was collected in Brazil in 1991 and identified by Terence D. Pennington (Royal Botanic Gardens Kew). The herbarium voucher associated with the sequenced plant is BROWP2036 and is deposited in the herbarium of the Royal Botanic Garden Edinburgh (Herbarium code: E).

### Nucleic acid extraction

The workflow for high molecular weight (HMW) DNA extraction at the Wellcome Sanger Institute (WSI) Tree of Life Core Laboratory includes a sequence of core procedures: sample preparation and homogenisation, DNA extraction, fragmentation and purification. Detailed protocols are available on protocols.io ( Denton *et al.*, 2023).

For sample homogenisation, leaf tissue was cryogenically disrupted using the Covaris cryoPREP ^®^ Automated Dry Pulverizer ( Narváez-Gómez *et al.*, 2023). HMW DNA was extracted using the Manual MagAttract protocol ( Strickland *et al.*, 2023b). HMW DNA was sheared into an average fragment size of 12–20 kb in a Megaruptor 3 system ( Todorovic *et al.*, 2023). Sheared DNA was purified by solid-phase reversible immobilisation, using AMPure PB beads to sample to eliminate shorter fragments and concentrate the DNA ( Strickland *et al.*, 2023a). The concentration of the sheared and purified DNA was assessed using a Nanodrop spectrophotometer and Qubit Fluorometer with a Qubit dsDNA High Sensitivity Assay kit. Fragment size distribution was evaluated by running the sample on the FemtoPulse system.

RNA was extracted from leaf tissue of drIngLaur1 in the Tree of Life Laboratory at the WSI using the RNA Extraction: Automated MagMax™ *mir*Vana protocol ( do Amaral *et al.*, 2023). The RNA concentration was assessed using a Nanodrop spectrophotometer and a Qubit Fluorometer using the Qubit RNA Broad-Range Assay kit. Analysis of the integrity of the RNA was done using the Agilent RNA 6000 Pico Kit and Eukaryotic Total RNA assay.

### Library preparation and sequencing

Pacific Biosciences HiFi circular consensus DNA sequencing libraries were constructed according to the manufacturers’ instructions. Poly(A) RNA-Seq libraries were constructed using the NEB Ultra II RNA Library Prep kit. DNA and RNA sequencing was performed by the Scientific Operations core at the WSI on Pacific Biosciences Sequel IIe (HiFi) and Illumina NovaSeq 6000 (RNA-Seq) instruments.

Hi-C data were generated from the leaf tissue of drIngLaur1 using the Arima-HiC v2 kit. Leaf tissue (–80 °C) was finely ground using cryoPREP ^®^ and then subjected to nuclei isolation using the Qiagen QProteome Kit. After isolation, the nuclei were fixed, and the DNA crosslinked using pure formaldehyde. The crosslinked DNA was then digested using a restriction enzyme master mix. The 5’-overhangs were filled in and labelled with a biotinylated nucleotide, followed by proximity ligation. The biotinylated DNA constructs were fragmented to a size of 400 to 600 bp using a Covaris E220 sonicator. The DNA was then enriched, barcoded, and amplified using the NEBNext Ultra II DNA Library Prep Kit, following the manufacturer's instructions. Hi-C sequencing was performed using paired-end sequencing with a read length of 150 bp on an Illumina NovaSeq 6000 instrument.

### Genome assembly, curation and evaluation

***Assembly***

The original assembly of HiFi reads was performed using HiCanu ( Nurk *et al.*, 2020). The Hi-C reads were mapped to the primary contigs using bwa-mem2 ( Vasimuddin *et al.*, 2019). The contigs were further scaffolded using the provided Hi-C data ( Rao *et al.*, 2014) in YaHS ( Zhou *et al.*, 2023) using the --break option. The scaffolded assemblies were evaluated using Gfastats ( Formenti *et al.*, 2022), BUSCO ( Manni *et al.*, 2021) and MERQURY.FK ( Rhie *et al.*, 2020). The organelle genomes were assembled using OATK ( Zhou, 2023).

***Curation***

The assembly was decontaminated using the Assembly Screen for Cobionts and Contaminants (ASCC) pipeline (article in preparation). Manual curation was primarily conducted using PretextView ( Harry, 2022), with additional insights provided by JBrowse2 ( Diesh *et al.*, 2023) and HiGlass ( Kerpedjiev *et al.*, 2018). Scaffolds were visually inspected and corrected as described by Howe *et al*. (2021). Any identified contamination, missed joins, and mis-joins were corrected, and duplicate sequences were tagged and removed. The process is documented at https://gitlab.com/wtsi-grit/rapid-curation (article in preparation).

***Evaluation of final assembly***

The final assembly was post-processed and evaluated using the three Nextflow ( Di Tommaso *et al.*, 2017) DSL2 pipelines: sanger-tol/readmapping ( Surana *et al.*, 2023a), sanger-tol/genomenote ( Surana *et al.*, 2023b), and sanger-tol/blobtoolkit ( Muffato *et al.*, 2024). The readmapping pipeline aligns the Hi-C reads using bwa-mem2 ( Vasimuddin *et al.*, 2019) and combines the alignment files with SAMtools ( Danecek *et al.*, 2021). The genomenote pipeline converts the Hi-C alignments into a contact map using BEDTools ( Quinlan & Hall, 2010) and the Cooler tool suite ( Abdennur & Mirny, 2020). The contact map is visualised in HiGlass ( Kerpedjiev *et al.*, 2018). This pipeline also generates assembly statistics using the NCBI datasets report ( Sayers *et al.*, 2024), computes *k*-mer completeness and QV consensus quality values with FastK and MERQURY.FK, and runs BUSCO ( Manni *et al.*, 2021) to assess completeness.

The blobtoolkit pipeline is a Nextflow port of the previous Snakemake Blobtoolkit pipeline ( Challis *et al.*, 2020). It aligns the PacBio reads in SAMtools and minimap2 ( Li, 2018) and generates coverage tracks for regions of fixed size. In parallel, it queries the GoaT database ( Challis *et al.*, 2023) to identify all matching BUSCO lineages to run BUSCO ( Manni *et al.*, 2021). For the three domain-level BUSCO lineages, the pipeline aligns the BUSCO genes to the UniProt Reference Proteomes database ( Bateman *et al.*, 2023) with DIAMOND ( Buchfink *et al.*, 2021) blastp. The genome is also split into chunks according to the density of the BUSCO genes from the closest taxonomic lineage, and each chunk is aligned to the UniProt Reference Proteomes database with DIAMOND blastx. Genome sequences without a hit are chunked with seqtk and aligned to the NT database with blastn ( Altschul *et al.*, 1990). The blobtools suite combines all these outputs into a blobdir for visualisation.

The genome assembly and evaluation pipelines were developed using nf-core tooling ( Ewels *et al.*, 2020) and MultiQC ( Ewels *et al.*, 2016), relying on the Conda package manager, the Bioconda initiative ( Grüning *et al.*, 2018), the Biocontainers infrastructure ( da Veiga Leprevost *et al.*, 2017), as well as the Docker ( Merkel, 2014) and Singularity ( Kurtzer *et al.*, 2017) containerisation solutions.

Table 4 contains a list of relevant software tool versions and sources.

**Table 4.** Software tools: versions and sources.

Software tool	Version	Source
BEDTools	2.30.0	https://github.com/arq5x/bedtools2
Blast	2.14.0	ftp://ftp.ncbi.nlm.nih.gov/blast/executables/blast+/
BlobToolKit	4.3.7	https://github.com/blobtoolkit/blobtoolkit
BUSCO	5.4.3 and 5.5.0	https://gitlab.com/ezlab/busco
bwa-mem2	2.2.1	https://github.com/bwa-mem2/bwa-mem2
Cooler	0.8.11	https://github.com/open2c/cooler
DIAMOND	2.1.8	https://github.com/bbuchfink/diamond
fasta_windows	0.2.4	https://github.com/tolkit/fasta_windows
FastK	427104ea91c78c3b8b8b49f1a7d6bbeaa869ba1c	https://github.com/thegenemyers/FASTK
Gfastats	1.3.6	https://github.com/vgl-hub/gfastats
GoaT CLI	0.2.5	https://github.com/genomehubs/goat-cli
HiCanu	2.2	https://github.com/marbl/canu
HiGlass	44086069ee7d4d3f6f3f0012569789ec138f42b84aa44357826c0b6753eb28de	https://github.com/higlass/higlass
Merqury.FK	d00d98157618f4e8d1a9190026b19b471055b22e	https://github.com/thegenemyers/MERQURY.FK
MultiQC	1.14, 1.17, and 1.18	https://github.com/MultiQC/MultiQC
NCBI Datasets	15.12.0	https://github.com/ncbi/datasets
Nextflow	23.04.0-5857	https://github.com/nextflow-io/nextflow
PretextView	0.2	https://github.com/sanger-tol/PretextView
OATK	0.2	https://github.com/c-zhou/oatk
samtools	1.16.1, 1.17, and 1.18	https://github.com/samtools/samtools
sanger-tol/genomenote	1.1.1	https://github.com/sanger-tol/genomenote
sanger-tol/readmapping	1.2.1	https://github.com/sanger-tol/readmapping
Seqtk	1.3	https://github.com/lh3/seqtk
Singularity	3.9.0	https://github.com/sylabs/singularity
TreeVal	1.0.0	https://github.com/sanger-tol/treeval
YaHS	1.1a.2	https://github.com/c-zhou/yahs

### Wellcome Sanger Institute – Legal and Governance

The materials that have contributed to this genome note have been supplied by a Darwin Tree of Life Partner. The submission of materials by a Darwin Tree of Life Partner is subject to the **‘Darwin Tree of Life Project Sampling Code of Practice’**, which can be found in full on the Darwin Tree of Life website here. By agreeing with and signing up to the Sampling Code of Practice, the Darwin Tree of Life Partner agrees they will meet the legal and ethical requirements and standards set out within this document in respect of all samples acquired for, and supplied to, the Darwin Tree of Life Project.

Further, the Wellcome Sanger Institute employs a process whereby due diligence is carried out proportionate to the nature of the materials themselves, and the circumstances under which they have been/are to be collected and provided for use. The purpose of this is to address and mitigate any potential legal and/or ethical implications of receipt and use of the materials as part of the research project, and to ensure that in doing so we align with best practice wherever possible. The overarching areas of consideration are:

- Ethical review of provenance and sourcing of the material
- Legality of collection, transfer and use (national and international)

Each transfer of samples is further undertaken according to a Research Collaboration Agreement or Material Transfer Agreement entered into by the Darwin Tree of Life Partner, Genome Research Limited (operating as the Wellcome Sanger Institute), and in some circumstances other Darwin Tree of Life collaborators.

## Data Availability

European Nucleotide Archive: *Inga laurina*. Accession number PRJEB65690; https://identifiers.org/ena.embl/PRJEB65690 ( Wellcome Sanger Institute, 2023). The genome sequence is released openly for reuse. The *Inga laurina* genome sequencing initiative is part of the Darwin Tree of Life (DToL) project. All raw sequence data and the assembly have been deposited in INSDC databases. Raw data and assembly accession identifiers are reported in Table 1.

## Author information

Members of the Wellcome Sanger Institute Tree of Life Management, Samples and Laboratory team are listed here: https://doi.org/10.5281/zenodo.12162482.

Members of Wellcome Sanger Institute Scientific Operations: Sequencing Operations are listed here: https://doi.org/10.5281/zenodo.12165051.

Members of the Wellcome Sanger Institute Tree of Life Core Informatics team are listed here: https://doi.org/10.5281/zenodo.12160324.

Members of the Tree of Life Core Informatics collective are listed here: https://doi.org/10.5281/zenodo.12205391.
