# Supplementary material for: The genome sequence of Inga laurina (Sw.) Willd
Source: Wellcome Open Res. 2024 Oct 7;9:567. [Version 1] doi: 10.12688/wellcomeopenres.23057.1 (PMC11558168; doi:10.12688/wellcomeopenres.23057.1)
Supplement: Peer review history

# Reviewer report: Reviewer response for version 1

**DOI:** 10.21956/wellcomeopenres.25393.r107661

**Published:** 2024-11-12

**Authors:** Jennifer A Kimball

**Affiliations:** 1 University of Minnesota, Saint Paul, Minnesota, USA

This is well-written manuscript presenting a genome assembly of a neotropical tree species, *Inga laurina*, and I applaud the work. The description of sequencing and subsequent analyses, in both the methods and results, are sufficient for indexing. This or future work would benefit from annotation of the defense responses found in the species.

Are sufficient details of methods and materials provided to allow replication by others?

Yes

Is the rationale for creating the dataset(s) clearly described?

Yes

Are the datasets clearly presented in a useable and accessible format?

Yes

Are the protocols appropriate and is the work technically sound?

Yes

Reviewer Expertise:

genomics and evolutionary genetics

I confirm that I have read this submission and believe that I have an appropriate level of expertise to confirm that it is of an acceptable scientific standard.

# Reviewer report: Reviewer response for version 1

**DOI:** 10.21956/wellcomeopenres.25393.r104034

**Published:** 2024-11-02

**Authors:** Marie Bolger

**Affiliations:** 1 Institute for Bio- and Geosciences, Forschungszentrum Jülich, Jülich, Germany

- This is a high-quality addition to the list of plant genomes sequenced.
  The paper is purely focused on providing a high-quality genome assembly (including Mitochondria and Chloroplast) of *Inga laurina* and in this, it has achieved its goal.  
- "Here we present one of three chromosomally complete, annotated genome sequences for *Inga*"
  Is this referring to the other two species (also by the same authors) *Inga oerstediana* Benth and *Inga leiocalycina* Benth?
  This information should be included in this manuscript.
- "...from 2.41 million reads, providing approximately 30-fold coverage"
  There is no mention of the genome size (estimated) - was there data available to indicate what length the genome was expected to be?  
- Details for RNA extraction and library prep and sequencing are provided in the manuscript but no detail for what the RNA was used for.
- Clearly the assumption is that it was used for structural gene annotation, but using RNA from just a single tissue for this purpose is unlikely to give optimal results.
- Gene (structural) annotation was performed by the 'Ensembl Rapid Release'. No details as to what this entailed or a referenced to it is provided.
- I would be interested in some detail of the 'Assembly Screen for Cobionts and Contaminants (ASCC) pipeline'.
  If the ASCC manuscript is not yet ready, details should be provided in this manuscript.

**Data availability:**

- Could you also provide a link here to the genome assembly and gene annotations in this section.
https://ftp.ensembl.org/pub/rapid-release/species/Inga_laurina/GCA_963855925.1/ensembl/ 
- "The average transcript length is 3,508.99. There are 1.45 coding transcripts per gene and 4.91 exons per transcript"
  These numbers are not particularly helpful without some context (are these numbers good for a plant in general?).
- Mercator4 provides a breakdown of how much a protein length deviates from the median length for each 'high quality' annotation.
- This information indicated that the proteome for *Inga laurina *has many proteins which are small.
https://www.plabipd.de/projects/Mercator4_output/inga_laurina_length.png

Are sufficient details of methods and materials provided to allow replication by others?

No

Is the rationale for creating the dataset(s) clearly described?

Partly

Are the datasets clearly presented in a useable and accessible format?

Yes

Are the protocols appropriate and is the work technically sound?

Yes

Reviewer Expertise:

genome assembly, structural gene annotation, functional genome annotation

I confirm that I have read this submission and believe that I have an appropriate level of expertise to confirm that it is of an acceptable scientific standard.
